# Less Social Support for Patients With COVID-19: Comparison With the Experience of Nurses

**DOI:** 10.3389/fpsyt.2021.554435

**Published:** 2021-02-01

**Authors:** Zhenyu Li, Jingwu Ge, Jianping Feng, Riyue Jiang, Qin Zhou, Xiaolin Xu, Yinbing Pan, Shijiang Liu, Bo Gui, Zhongyun Wang, Bin Zhu, Yimin Hu, Jianjun Yang, Rong Wang, Dongan Su, Kenji Hashimoto, Meiling Yang, Chun Yang, Cunming Liu

**Affiliations:** ^1^Department of Anesthesiology and Perioperative Medicine, The First Affiliated Hospital of Nanjing Medical University, Nanjing, China; ^2^Department of Ultrasound Imaging, Renmin Hospital of Wuhan University, Wuhan, China; ^3^Department of Anesthesiology, Tongji Medical College, Tongji Hospital, Wuhan, China; ^4^Department of Critical Care Medicine, The Third Affiliated Hospital of Soochow University, Suzhou, China; ^5^Department of Anesthesiology, The Second Affiliated Changzhou People's Hospital of Nanjing Medical University, Changzhou, China; ^6^Department of Anesthesiology, The First Affiliated Hospital of Zhengzhou University, Zhengzhou, China; ^7^Department of Nursing, The First Affiliated Hospital of Nanjing Medical University, Nanjing, China; ^8^Department of Rehabilitation and Pain Medicine, 904th Hospital of The Joint Logistics Support Force, People's Liberation Army, Changzhou, China; ^9^Division of Clinical Neuroscience, Chiba University Center for Forensic Mental Health, Chiba, Japan

**Keywords:** COVID-19, social support, medical staff, depression, anxiety

## Abstract

**Context:** Since December 2019, more than 80,000 patients have been diagnosed with coronavirus disease 2019 (COVID-19) in China. Social support status of COVID-19 patients, especially the impact of social support on their psychological status and quality of life, needs to be addressed with increasing concern.

**Objectives:** In this study, we used social support rating scale (SSRS) to investigate the social support in COVID-19 patients and nurses.

**Methods:** The present study included 186 COVID-19 patients at a Wuhan mobile cabin hospital and 234 nurses at a Wuhan COVID-19 control center. Responses to a mobile phone app-based questionnaire about social support, anxiety, depression, and quality of life were recorded and evaluated.

**Results:** COVID-19 patients scored significantly lower than nurses did on the Social Support Rating Scale (SSRS). Among these patients, 33.9% had anxiety symptoms, while 23.7% had depression symptoms. Overall SSRS, subjective social support scores and objective support scores of patients with anxiety were lower than those of patients without anxiety. This result was also found in depression. In addition, all dimensions of social support were positively correlated with quality of life. Interestingly, in all dimensions of social support, subjective support was found to be an independent predictive factor for anxiety, depression, and quality of life, whereas objective support was a predictive factor for quality of life, but not for anxiety and depression via regression analysis.

**Conclusion:** Medical staffs should pay attention to the subjective feelings of patients and make COVID-19 patients feel respected, supported, and understood from the perspective of subjective support, which may greatly benefit patients, alleviate their anxiety and depression, and improve their quality of life.

## Introduction

Since December 2019, more than 80,000 individuals have been diagnosed with coronavirus disease 2019 (COVID-19), and more than 3,000 patients have died during the spread of COVID-19. China has immediately and decisively taken active and effective measures to support the anti-COVID-19 effort in Wuhan. To date, during the outbreak of the COVID-19 epidemic in Wuhan, ~42,000 medical staffs have gone to Wuhan City and Hubei Province to provide medical assistance, where COVID-19 patients were isolated from their families and friends for treatment, possibly affecting their social support status. More importantly, the medical staffs were also isolated from the rest of society in order to provide medical service; thus, to some extent, their social support status would also be affected during their work with COVID-19 patients.

COVID-19 patients have higher levels of depression, anxiety and stress than healthy controls and the emotions experienced by COVID-19 patients are often shock, fear, despair, hope, and boredom (1). In addition, under the epidemic, healthcare workers in various countries are also suffering from different levels of psychological distress (2). Increasingly, evidence shows that social support is positively related to psychological health and quality of life, that is, enhancing social support would improve the mental health and quality of life of the recipients (3–5). It has been reported that social support has a protective effect on mental health; it plays a direct role via social relationships and exerts an indirect effect through the inhibition of excessive stress (6, 7). Several lines of evidence indicate that social support can provide beneficial effects to reduce the risk of depression in children, adolescents, young adults, middle-aged people, the elderly, and healthcare workers (8–11). Similarly, regarding anxiety assessment, a large number of studies suggest that the anxiety score is inversely related to social support (12). In other words, social support also has a protective effect against anxiety, and a low social support score can be used to predict the incidence of anxiety (13, 14). Therefore, during the spread and control of COVID-19, it is particularly important to pay attention to social support for the general public.

The purpose of this study was to observe and compare the social support received by COVID-19 patients and nurses as well as to explore the association between anxiety, depression, and social support for measuring the predictive factors of depression and anxiety in both groups. The results will shed light on how to provide sufficient social support for COVID-19 patients and medical staffs during the effort to control COVID-19, as well as objective evidence for the prevention and treatment of anxiety, depression, and other psychological problems, ultimately improving quality of life.

## Methods

### Settings and Participants

For this descriptive study, we used a mobile phone app-based questionnaire survey during the COVID-19 pandemic from February 17, 2020 to Mar 17, 2020. The Ethics Committee of the First Affiliated Hospital of Nanjing Medical University approved this study (approval number: 2020-SR-111). We observed COVID-19 patients treated at the Wuhan Sports Center Mobile Cabin Hospital and frontline nurses working to control COVID-19 in Wuhan. Owing to the fact that the investigation was conducted during the COVID-19 pandemic, the isolation policy at the time called for reduced face-to-face contact and communication, as well as the avoidance of large gatherings and activities. Therefore, an anonymous questionnaire was constructed using a mobile app called Sojump (www.sojump.com) and sent to individuals via WeChat after obtaining informed consent as we previously reported (15). According to Kendall's sample size estimation method, the sample size is at least 5 times than that of the variable (16). Given that the loss of samples during the study (loss of 10%), a total of 420 individuals, including 186 COVID-19 patients and 234 nurses, filled in the questionnaire. Individuals who have the pre-existing psychiatric abnormalities have been excluded and all the nurses with work license are full-time employees in medical institutions.

### Assessment of Patient-Reported Outcomes

Demographic data, including gender, age, educational background, marriage status, habitation, employment, income, tobacco and alcohol usage, and comorbidities, were recorded. Social support was assessed using the Social Support Rating Scale (SSRS), which is currently widely used to measure the social support for the general public, patients, and medical staff. The SSRS used in our study was created primarily for the Chinese population (17). It includes three dimensions (subjective social support, objective social support, and utilization of support). The total social support score is the sum of the score of the three dimensions, and higher scores indicate higher levels of perceived social support.

Anxiety and depression symptoms were assessed using the Hospital Anxiety and Depression Scale (HADS) (18). The HADS is a self-rated scale and consists of 14 items, seven for anxiety (HADS-A) and seven for depression (HADS-D), with a 4-point scale (ranging from 0 = not at all to 3 = very much indeed). Both the HADS-A and HADS-D scores range from 0 to 21. A patient with a HADS-A score or HADS-D score ≥ 8 is identified as having anxiety or depression. The HADS is a reliable instrument for detecting states of depression and anxiety among hospital patients, and it is also a valid measurement of the severity of mental disorders.

Quality of life was assessed by using the World Health Organization Quality of Life instrument (WHOQOL-BREF), which consists of 26 questions (19). It includes two separate items used to evaluate the general quality of life (question 1) and satisfaction with one's state of health (question 2), while the other 24 questions involve the following four domains: physical health, psychological health, social relationships, and environment. In this study, the total quality of life score is the sum of the scores of four domains. Higher WHOQOL scores indicate a better quality of life.

### Statistical Analysis

For this study, continuous and normally distributed variables were used for the means and the standard error of the mean, and an independent sample *t*-test was conducted to assess the two groups' differences. Abnormally distributed data were described using the median and interquartile range (IQR: 25–75%), whereas the Mann–Whitney *U*-test was used to assess two group differences. Descriptive statistics involved frequencies (%) for categorical variables, and the chi-square test and Fisher's exact-test were used to assess the two groups' differences. Spearman's rank correlation analysis was used to evaluate the relationship between the two variables. Moreover, multiple linear regression and binary logistics regression analyses were used to determine the risk factors associated with quality of life, anxiety, and depression. Results were presented as odds ratio (OR) and 95% confidence intervals (CIs). Receiver operating characteristic (ROC) was used to evaluate the performance of the regression model. Data were considered statistically significant when *P* < 0.05. Analyses were performed using Statistical Package for the Social Sciences (SPSS) software version 20.0 (IBM Co. LTD, Chicago, IL, USA).

## Results

### General Characteristics of COVID-19 Patients and Nurses

A total of 420 individuals (i.e., 186 COVID-19 patients and 234 nurses) were enrolled in the study. Table 1 shows the general characteristics of COVID-19 patients and nurses (partial data on the general characteristics of 234 nurses are available in our previously published study) (15). Gender, age, educational background, and marriage status all were statistically significant among COVID-19 patients and nurses (*P* < 0.001).

**Table 1:** General characteristics of COVID-19 and nurses.

	**COVID-19 (*n* = 186)**	**Nurses (*n* = 234)**	***P-*value**
**Gender, %**			<0.001^a^
Male	108 (58.06)	28 (11.97)	
Female	78 (41.94)	206 (88.03)	
**Age, median (IQR), year**	38(31–48)	29.5 (26-34)	<0.001^b^
**Education background, %**			<0.001^a^
College degree or lower	111 (59.68)	43 (18.38)	
Bachelor or higher degree	75 (40.32)	191 (81.62)	
**Marriage, %**			<0.001^a^
Unmarried	28 (15.05)	123 (52.56)	
Married	143 (76.88)	105 (44.87)	
Divorce or others	15 (8.06)	6 (2.56)	
**Habitation, %**		NA	
Urban	156 (83.87)		
Rural area	30 (16.13)		
**Employment, %**		NA	
Yes	174 (93.55)		
No	12 (6.45)		
**Income/person/year, %, RMB**		NA	
<15,000	27(14.52)		
15,000–33,000	43 (23.12)		
>33,000	116 (62.37)		
**Tobacco usage, %**		NA	
Yes	142 (76.34)		
No	44 (23.66)		
**Drinking history, %**		NA	
Yes	131 (70.43)		
No	55 (29.57)		
**Comorbidities, %**		NA	
Yes	26 (13.98)		
No	160 (86.02)		

*COVID-19, corona virus disease 2019; IQR, interquartile range; NA, not applicable*.

a *Chi-square-test*.

b *Mann–Whitney U-test*.

**Figure 1:**
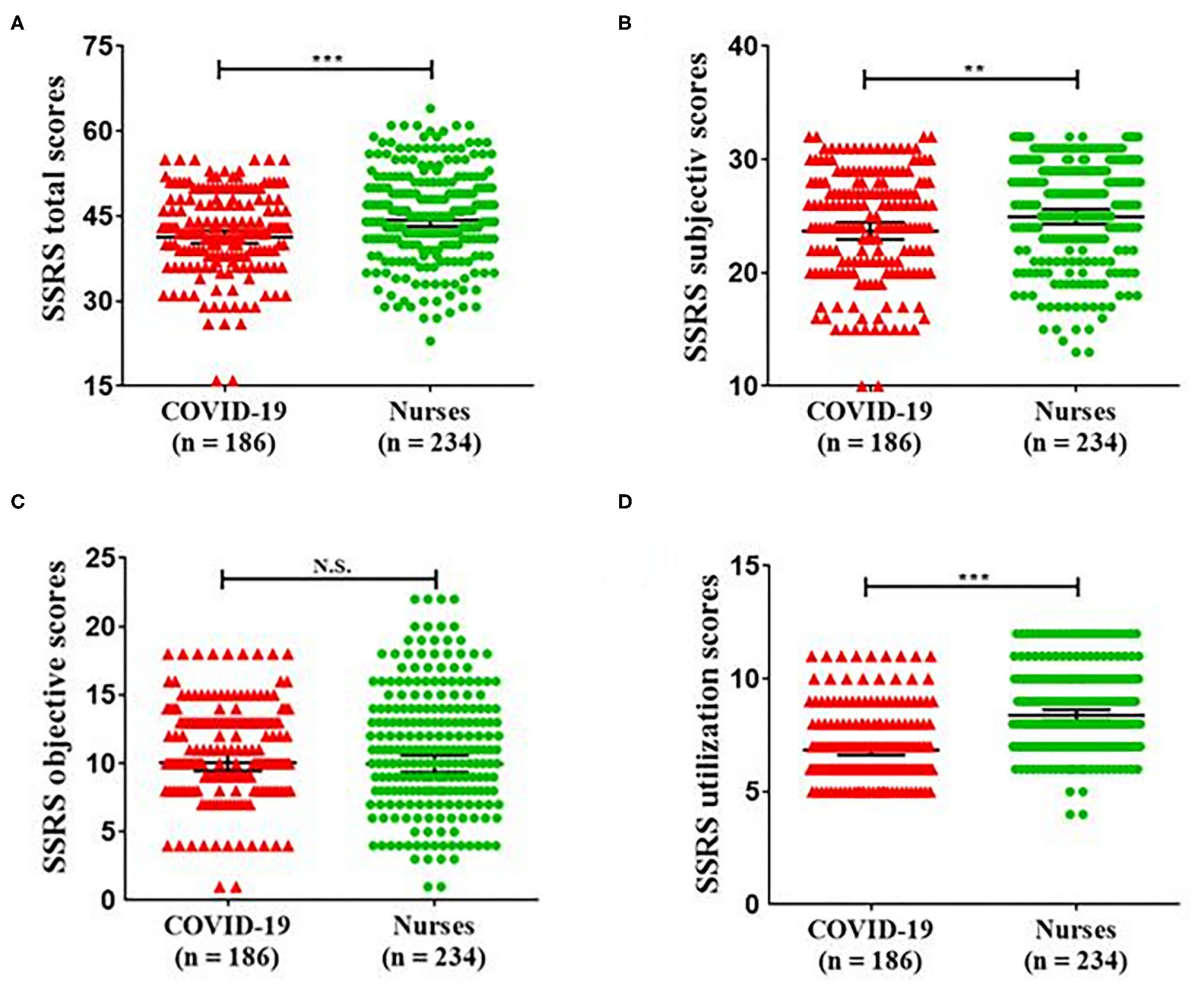
Scores for SSRS total **(A)**, SSRS subjective **(B)**, SSRS objective **(C)**, and SSRS utilization **(D)** between COVID-19 patients and nurses. SSRS total scores (*P* = 0.008); SSRS subjective scores (*P* = 0.023); SSRS objective scores (*P* = 0.986); SSRS utilization scores (*P* < 0.001). COVID-19, coronavirus disease 2019; SSRS, Social Support Rating Scale. **p < 0.05, ***p < 0.01.

The total SSRS score consists of subjective support, objective support, and support utilization. More importantly, the total SSRS scores for COVID-19 patients (42; IQR = 38–48) were significantly lower as compared with those of nurses (45; IQR = 40–51; *P* < 0.001). No significant difference was found for objective support between COVID-19 patients (10; IQR = 8–13) and nurses (11; IQR = 8–14; *P* = 0.805). On the contrary, the subjective support scores were significantly lower in COVID-19 patients (24; IQR = 21–28) as compared with those of nurses (26; IQR = 22–30; *P* = 0.007). The support utilization scores were also significantly lower in COVID-19 patients (7; IQR = 6–8) than those of nurses (8; IQR = 7–10; *P* < 0.001). The details are displayed in Figure 1 and Table 2.

**Table 2:** Basic conditions of social support, anxiety, depression and quality of life in COVID-19.

	**COVID-19 (*n* = 186)**
**SSRS-Social support, median (IQR)**	
Total support	42 (38–48)
Subjective support	24 (21–28)
Objective support	10 (8–13)
Utilization of support	7 (6–8)
**HADS-Anxiety**	
Yes, %	63 (33.9)
No, %	143 (66.1)
Median (IQR)	6 (4–8)
**HADS-Depression**	
Yes, %	44 (23.7)
No, %	142 (76.3)
Median (IQR)	5 (2–7)
**WHO-Quality of life, median (IQR)**	
Total quality of life	58.5 (53.9–62.7)
Physical health	15.4 (14.3–16.7)
Psychological health	14.7 (13.3–16)
Social relationship	14.7 (13.3–16)
Environment	14.5 (13–15.5)

*COVID-19, corona virus disease 2019; HADS, hospital anxiety and depression scale; IQR, interquartile range; SSRS, social support revalued scale; WHO, world health organization*.

### Comparison of SSRS in COVID-19 Patients With or Without Anxiety/Depression Symptoms

A total of 186 COVID-19 patients were enrolled in the study. The anxiety score was 6 [4–8, median (IQR)], and 63 (33.9%) COVID-19 patients had anxiety symptoms according to the HADS evaluation (Table 2). The SSRS rendered the following results:

- total support, including anxiety (39; IQR = 36–43) and non-anxiety (43; IQR = 40–50; *P* < 0.001), which showed a statistical difference;
- subjective support, including anxiety (23; IQR = 20–26) and non-anxiety (26; IQR = 22–29; *P* < 0.001), also demonstrating a statistical difference;
- objective support, including anxiety (10; IQR = 8–12) and non-anxiety (11; IQR = 9–13; *P* = 0.010), exhibiting significant difference; and

All showed a significant difference between COVID-19 patients with and without anxiety symptoms. Intriguingly, support utilization (anxiety: 6; IQR = 6–7 and non-anxiety: 7; IQR = 6–8; *P* = 0.122) failed to show a statistical difference (Table 3).

**Table 3:** Comparison of social support in COVID-19 patients with or without anxiety/depression symptoms.

	**HADS-Anxiety**		***P***	**HADS-Depression**		***P***
	**≥8 score**	**<8 score**		**≥8 score**	**<8 score**	
**SSRS, median (IQR)**						
Total support	39 (36–43)	43 (40–50)	<0.001	36 (31.3–42.8)	43 (39–48.5)	<0.001
Subjective support	23 (20–26)	26 (22–29)	<0.001	21 (17–25.5)	26 (22–29)	<0.001
Objective support	10 (8–12)	11 (9–13)	0.010	8 (7–12)	11 (9–13)	0.001
Utilization of support	6 (6–7)	7 (6–8)	0.122	6 (5.3–7)	7 (6–8)	0.149

*COVID-19, corona virus disease 2019; HADS, hospital anxiety and depression scale; SSRS, social support revalued scale; IQR, interquartile range*.

As for depression symptoms, the depression score was 5 (2–7) (median [IQR]), and 44 (23.7%) COVID-19 patients had depression symptoms (Table 2). The SSRS results are as follows:

- total support, including depression (36; IQR = 31.3–42.8) and non-depression (43; IQR = 39–48.5; *P* < 0.001);
- subjective support, including depression (21; IQR = 17–25.5) and non-depression (26; IQR = 22–29; *P* < 0.001); and
- objective support, including depression (8; IQR = 7–12) and non-depression (11; IQR = 9–13; *P* = 0.001).

All showed a significant difference between COVID-19 patients with and without depression symptoms. Intriguingly, support utilization (depression: 6; IQR = 5.3–7 and non-depression: 7; IQR = 6–8; *P* = 0.149) failed to show a statistical difference (Table 3).

### Correlation Between Anxiety, Depression, Quality of Life, and SSRS in COVID-19 Patients

We found that the anxiety scores were negatively associated with the total SSRS scores (*R* = −0.268; *P* < 0.001), subjective support (*R* = −0.264; *P* < 0.001) and objective support (*R* = −0.195; *P* = 0.008). The depression scores were negatively associated with all dimensions of social support (total SSRS: *R* = −0.458, *P* < 0.001; subjective support: *R* = −0.427, *P* < 0.001; objective support: *R* = −0.290, *P* < 0.001; support utilization: *R* = −0.211, *P* = 0.004). Similarly, the total quality of life scores were positively associated with all dimensions of social support (total SSRS: *R* = 0.315, *P* < 0.001; subjective support: *R* = 0.298, *P* < 0.001; objective support: *R* = −0.203, *P* = 0.005; support utilization: *R* = 0.265, *P* < 0.001) (Table 4).

**Table 4:** Correlation coefficient matrix for social support, anxiety, depression and quality of life in COVID-19 patients.

	**1**	**2**	**3**	**4**	**5**	**6**	**7**	**8**	**9**	**10**	**11**
1. Total social support	1.000										
2. Subjective support	0.822^**^	1.000									
3. Objective support	0.716^**^	0.320^**^	1.000								
4. Utilization of support	0.253^**^	0.041	0.082	1.000							
5. Anxiety	−0.268^**^	−0.264^**^	−0.195^**^	−0.113	1.000						
6. Depression	−0.458^**^	−0.427^**^	−0.290^**^	−0.211^**^	−0.724^**^	1.000					
7. Total quality of life	0.351^**^	0.298^**^	0.203^**^	0.265^**^	−0.455^**^	−0.599^**^	1.000				
8. Physical health	0.285^**^	0.196^**^	0.194^**^	0.250^**^	−0.499^**^	−0.567^**^	0.879^**^	1.000			
9. Psychological health	0.352^**^	0.285^**^	0.182^*^	0.266^**^	−0.372^**^	−0.609^**^	0.823^**^	0.641^**^	1.000		
10. Social relationship	0.340^**^	0.406^**^	0.121	0.144	−0.194^**^	−0.385^**^	0.787^**^	0.557^**^	0.626^**^	1.000	
11. Environment	0.283^**^	0.201^**^	0.207^**^	0.242^**^	−0.358^**^	−0.469^**^	0.799^**^	0.692^**^	0.596^**^	0.463^**^	1.000

*COVID-19, corona virus disease 2019*.

* *P < 0.05*,

** *P < 0.01*.

### Predictive Factors of Anxiety, Depression, and Quality of Life

A binary logistics regression analysis was performed to evaluate the predictive factors for anxiety and depression, whereas a multiple linear regression analysis was carried out to evaluate predictors of quality of life. Interestingly, the results showed that subjective support—but not objective support or support utilization—was a predictor of anxiety (OR = 0.729; 95% CI = 0.648–0.820; *P* < 0.001) and depression (OR = 0.745; 95% CI = 0.668–0.831; *P* = 0.004). Furthermore, anxiety was predicted by gender (OR = 13.259; 95% CI = 4.164–42.215; *P* < 0.001), age (OR = 1.083; 95% CI = 1.028–1.141; *P* = 0.003), employment (OR = 0.099; 95% CI =0.014–0.671; *P* = 0.018), income (OR = 2.110; 95% CI =1.085–4.101; *P* = 0.028) and comorbidities (OR = 0.057; 95% CI = 0.015–0.221; *P* < 0.001), whereas gender (OR = 5.937; 95% CI = 2.229–15.808; *P* < 0.001) and income (OR =2.234; 95% CI = 1.183–4.221; *P* = 0.013) were also a predictive factor for depression (Table 5). Results of ROC analysis showed that the strongest predictor for anxiety was the age (AUC: 0.674; 95% CI: 0.443–0.619) while for depression was the gender (AUC: 0.568; 95% CI: 0.470–0.665). Quality of life was predicted by gender (OR = −3.524; 95% CI = −5.954–−1.680; *P* = 0.001), drinking history (OR = −2.955; 95% CI = −5.825–−1.160; *P* = 0.034), comorbidities (OR =4.682; 95% CI = 0.284–0.698; *P* = *P* < 0.001), and objective support (OR = 2.918; 95% CI =0.277–1.432; *P* = 0.004) (Table 5).

**Table 5:** Regression analysis of anxiety, depression and quality of life in COVID-19 patients.

	**Anxiety^a^**			**Depression^b^**			**Quality of life^c^**		
	**β**	**OR (95% CI)**	***P***	**β**	**OR (95% CI)**	***P***	**β**	**OR (95% CI)**	***P***
Gender	2.585	13.259 (4.164, 42.215)	** <0.001**	1.781	5.937 (2.229, 15.808)	**<0.001**	−3.817	−3.524 (−5.954, −1.680)	**0.001**
Age	0.080	1.083 (1.028, 1.141)	**0.003**						
Habitation	−22.237	0.000 (0.000, N.S.)	0.997	−21.769	0.000 (0.000, N.S.)	0.997			
Employment	−2.316	0.099 (0.014, 0.671)	**0.018**						
Income	0.746	2.110 (1.085, 4.101)	**0.028**	0.804	2.234 (1.183, 4.221)	**0.013**			
Drinking history							−3.493	−2.955 (−5.825, −1.160)	**0.034**
Comorbidities	−2.869	0.057 (0.015, 0.221)	**<0.001**				3.133	2.142 (0.246, 6.019)	**<0.001**
Subjective support	−0.316	0.729 (0.648, 0.820)	**<0.001**	−0.294	0.745 (0.668, 0.831)	**0.004**	0.491	4.682 (0.284, 0.698)	**<0.001**
Objective support							0.855	2.918 (0.277, 1.432)	**0.004**

*Variables excluded by regression model*:

a *education background, marriage, tobacco usage, alcohol usage, objective support, utilization of support*;

b *age, education background, marriage, employment, tobacco usage, alcohol usage, comorbidities, objective support, utilization of support*;

c *age, education background, marriage, habitation, employment, income, tobacco usage, utilization of support*.

*COVID-19, corona virus disease 2019; OR, odds ratio. CI, confidence interval*.

*Bold values represent p < 0.05 (significant)*.

## Discussion

In the present study, social support, including subjective support, objective support, and support utilization among patients with COVID-19 were significantly lower than among nurses. In addition, we found that 33.9% of the 186 COVID-19 patients had anxiety, and 23.7% had depression. Furthermore, the social support of COVID-19 patients with anxiety or depression was significantly lower than that of those without anxiety or depression. It is noteworthy that, although the three dimensions of social support are related to anxiety, depression, and the quality of life of patients with COVID-19, subjective support serves as an independent predictor for anxiety, depression, and quality of life. It can be seen that the social support of patients with COVID-19 is lower, and their anxiety and depression are more serious. Compared with objective support and support utilization, subjective support is the key factor that affects patients' psychological status and quality of life. Moreover, it is well-recognized that anxiety, depression, and quality of life are highly related to gender. Female patients with COVID-19 are more likely to be anxious and/or depressed with lower quality of life, and that the presence of chronic comorbidities (e.g., diabetes, hypertension, etc.) also makes COVID-19 patients more prone to anxiety and lower quality of life. Collectively, these results suggest that female patients with COVID-19 and other chronic diseases are more likely to have mental disorders.

It is well-known that, during the spread of COVID-19, China has taken active and effective measures to establish more than 10 mobile cabin hospitals to treat patients effectively and quickly control the epidemic. Common people have given strong response and support to the government's rapid and effective measures. Meanwhile, the patients left their families and friends for treatment in isolation in the mobile cabin hospitals. The unfamiliar living environment and inability to contact family and friends may be the possible reasons underlying the problems of social support in COVID-19 patients (20, 21). In addition, COVID-19 is not well-understood, and its pathogenesis has not yet been determined. This lack of knowledge of COVID-19 and worries about the health and living conditions of family and friends may induce the onset of fear, anxiety, and depression. In the process of providing medical treatment for patients with COVID-19, we should pay more attention to the social support and psychological status of these patients while solving problems in a timely manner and taking active and effective countermeasures.

Social support is highly related to patients' psychological status and quality of life (13), as our results also confirm: the lower the social support, the more serious the anxiety and depression symptoms; by contrast, the higher the social support, the higher the quality of life. It has been reported that better psychological conditions improve patients' treatment compliance and immunity (22, 23), a great advantage for COVID-19 patients. In addition, social support can be divided into three categories: subjective social support (emotional support), objective support (visible or actual support), and support utilization (individual response to external support) (17). It must be emphasized that subjective social support is the main factor that affects the mental state and quality of life of patients with COVID-19. Subjective social support is closely related to the individual's subjective feelings. It refers to the emotional experience and satisfaction that an individual is respected, supported, and understood in the society. Therefore, we should focus on improving the strength of subjective support for patients, as well as encouraging and comforting patients in the treatment process (24). Although objective support and support utilization are not independent factors affecting COVID-19 patients, they still cannot be ignored.

Increasingly, evidence shows that gender is an independent predictor for mental disorders (25, 26). Women with COVID-19 are more likely to have anxiety and depression symptoms during the spread of epidemics, which may be related to the fact that women are more sensitive to personal growth and interpersonal relationships than men (27, 28). In addition, chronic diseases have a deleterious psychological impact on patients (29). Our study also suggests that patients with comorbidities are more likely to suffer from anxiety and lower quality of life. Therefore, we should pay more attention to female patients with COVID-19 and actively intervene to help these patients avoid serious psychological problems.

We previously observed the status of vicarious traumatization (VT) in nurses and general public, but not in COVID-19 patients (15), since VT could only be adopted to evaluate the non-patient, especially the rescuers or caregivers. Very recently, a study reported the psychological status of medical staffs via the scores of Patient Health Questionnaire-9, which is a scale only evaluated for depression symptoms (30). However, in the present study, we majorly focused on the social support and its relationship with anxiety, depression, and quality of life of COVID-19 patients in this study. In addition, during COVID-19 isolation, internet-based education, training, and treatment can be used to receive social support, reduce anxiety and depression, making psychotherapy not only more convenient, but also more cost-effective (31, 32).

This study has several limitations. First, this study has a small sample size, although there are more patients with COVID-19, our study follows the voluntary principles. Second, the medical staffs enrolled in this study consisted of nurses only, not included physicians. Larger-number of medical workers should be included in future studies. Third, this is a single-center and descriptive cross-sectional study and mainly used self-reported questionnaires to measure psychosocial symptoms, while the gold standard for establishing psychosocial diagnosis involved clinical interviews and functional neuroimaging (33). Therefore, a large longitudinal study is necessary to further determine the causal linkage between the social support and mental health.

In conclusion, the results suggest that COVID-19 patients suffer from a lack of social support, which may exacerbate their psychological problems. Therefore, early intervention should be implemented to improve COVID-19 patients' social support to relieve their psychological problems, which would aid them in their recovery from COVID-19.

## Data Availability Statement

The raw data supporting the conclusions of this article will be made available by the authors, without undue reservation.

## Ethics Statement

The studies involving human participants were reviewed and approved by The Ethics Committee of The First Affiliated Hospital of Nanjing Medical University. The patients/participants provided their written informed consent to participate in this study.

## Author Contributions

ZL, JG, CY, and CL: concept and design. ZL and CY: drafting of the manuscript. ZL, JF, MY, CY, and CL: critical revision of the manuscript. ZL, JG, and CY: statistical analysis. MY, CL, and CY: supervision. All authors: acquisition, analysis, and interpretation of data.

## Conflict of Interest

The authors declare that the research was conducted in the absence of any commercial or financial relationships that could be construed as a potential conflict of interest.
